# Can Learning a Foreign Language Foster Analytic Thinking?—Evidence from Chinese EFL Learners' Writings

**DOI:** 10.1371/journal.pone.0164448

**Published:** 2016-10-14

**Authors:** Jingyang Jiang, Jinghui Ouyang, Haitao Liu

**Affiliations:** 1 Department of Linguistics, Zhejiang University, Hangzhou, 310058, China; 2 Ningbo Institute of Technology, Zhejiang University, Ningbo, CN-315100, China; University of Zurich, SWITZERLAND

## Abstract

Language is not only the representation of thinking, but also shapes thinking. Studies on bilinguals suggest that a foreign language plays an important and unconscious role in thinking. In this study, a software—Linguistic Inquiry and Word Count 2007—was used to investigate whether the learning of English as a foreign language (EFL) can foster Chinese high school students’ English analytic thinking (EAT) through the analysis of their English writings with our self-built corpus. It was found that: (1) learning English can foster Chinese learners’ EAT. Chinese EFL learners’ ability of making distinctions, degree of cognitive complexity and degree of thinking activeness have all improved along with the increase of their English proficiency and their age; (2) there exist differences in Chinese EFL learners’ EAT and that of English native speakers, i. e. English native speakers are better in the ability of making distinctions and degree of thinking activeness. These findings suggest that the best EFL learners in high schools have gained native-like analytic thinking through six years’ English learning and are able to switch their cognitive styles as needed.

## Introduction

Language is closely related to thought. They intricately intertwine with each other. Language is not only the representation of thinking, but also shapes thinking. Different people have different thinking patterns. Analytic thinking involves “a detachment of the object from its context and a focus on attributes of the object, whereas holistic thinking involves an orientation to the context or field as a whole” [1]. Cultural differences in thinking styles reveal that East Asian societies are characterized by holistic thinking and Western societies by analytic thinking [1]. East cultures view things as a whole, while western cultures focus on the units constituting the things [2]. In terms of the language structure of Chinese and English, Niu [3] explored the different thinking patterns preferred by different language users and found that Chinese prefer holistic thinking and lay emphasis on the whole, while Americans prefer analytic thinking and emphasize individual dependence. According to the weak version of Sapir-Whorf hypothesis, the structure of a language influences thought and certain kinds of non-linguistic behavior by affecting the ways in which its respective speakers conceptualize their worldviews, or otherwise influences their cognitive processes [4].

In recent studies, researchers started to focus on the people who speak multiple languages. Numerous researches have shown that bilingualism has cognitive benefits on the bilinguals beyond the acquisition of a foreign language (for more details, see [5,6]). There is no evidence that bilingualism affects intelligence, but a large number of important and prominent findings showed bilingual advantages in executive control in nonverbal control tasks that have been observed and verified in both children and adults [7–12]. Besides the positive influences on executive function, there is abundant evidence of other cognitive benefits of bilingualism, such as in creativity (expressed in divergent thinking or flexibility of thought) [13–16] and working memory performance [17,18]. To explore at what point in emerging bilingualism the bilingual advantages first appear, Sullivan *et al*. [19] investigated the effect of early second-language training on executive control. In their study, monolingual English-speaking students (17–32 years old) were tested on tasks before and after 6 months’ Spanish instruction. The evidence from ERP indicated that early stage second–language learning improves students’ executive control. In a longitudinal study [18] of memory advantages of bilinguals, monolinguals of Swedish and bilinguals (35–70 years old) were tested on episodic memory recall, verbal letter and categorical fluency during the trajectory of life. Results showed that bilinguals outperformed monolinguals both in episodic memory recall and letter fluency.

The above researches have revealed how bilingualism brought cognitive benefits (executive control, working memory, divergent thinking) to both children and adults, but few researches have touched upon the possible changes that could have brought to teenagers’ thinking style by foreign language learning. Moreover, the above studies mainly focus on subjects whose L1 (for monolinguals) and/or L2 (for bilinguals) are both alphabetic languages (English, Spanish, Swedish, French etc.), whose differences in languages are not as great as those between Chinese (an ideographic language) and English. Junior and senior high school period (K7-12, 12–18 years old) is the critical phase of learning English as a foreign language for Chinese students. They begin to learn English systematically after entering junior high school (K7) and gradually master English vocabulary, listening, speaking, reading and writing skills within six years, during which their thinking abilities are also at the critical developmental stage. Researchers have long been focusing on how to promote language proficiency, but unfortunately ignoring the other functions of English learning, such as its influences on thinking abilities, especially analytic thinking.

A number of studies have been conducted on the importance of analytic thinking [20] and how to foster analytic thinking [21, 22], many of which focus on analytic thinking, as well as holistic thinking from the perspective of cultural psychology. By assessing the subjects on the Analysis-Holism Scale (AHS), Choi *et al*. [23] found that the Koreans, belonging to Asian cultures, got higher scores on the AHS than the Americans, belonging to western cultures, and the Koreans displayed a more holistic pattern of performances on tasks. Another research [24] on analytic thinking and holistic thinking investigated how cultural differences in cognition posed a challenge to the management of information, and it found that Malaysians with higher scores on AHS than Americans remembered more situational information.

Nevertheless, no specific work has been done on the development of analytic thinking of Chinese students belonging to Asian cultures and characterized by holistic thinking from the perspective of bilingualism or foreign language acquisition, which might shed light on the positive influences of English learning on their development of analytic thinking. Thus, the present research on the possibility of fostering analytic thinking of Chinese high school students through English learning is meaningful and representative, because Chinese speakers can be the representative of holistic thinking of East Asians (such as the Chinese, Koreans, and Japanese) [25]. Before we proceed, it is necessary to clarify two different kinds of analytic thinking as far as we can see. One refers to Chinese students’ general analytic thinking (GAT), which might not be readily measured through their writings in English. As opposed to GAT, the other is the analytic thinking that Chinese learners gain through English learning, which we define as English analytic thinking (EAT). This EAT can be directly measured through their writings in English, even though its acquisition may be unconscious and implicit. This EAT is the focus of our present research. In the case of bilinguals, Chinese as their native language and English as their foreign language, can they, Chinese EFL learners characterized by holistic thinking, foster and improve their EAT through learning English? How will the Chinese learners’ EAT, which seems inherent among English native speakers, fluctuate along with the improvement of their English language proficiency? To what extent can their EAT be developed? All these thoughts boil down to two research questions:

Question 1. Can learning English foster Chinese EFL learners’ EAT? If yes, in what way?
Question 2. To what extent has Chinese EFL learners’ EAT developed?

## Method and Materials

This study tries to answer two research questions. Detailed information about the study’s method and materials, including participants, materials, procedures and data analysis are described as follows.

### Participants

Participants are 436 Chinese high school students from ten high schools in Zhejiang Province, China, who are scattered in different cities. We obtained the verbal approval from the students and their parents through high school teachers, who informed the students that their compositions would be used for academic purpose and their personal information would not be revealed in any fashion. The teachers told the students the assigned topics (designed by the researchers) and asked them to finish the compositions in the classroom within the limited time. Either the original compositions or the photocopied ones were then sent to us by the teachers. Participants’ basic information, including the number and the age of each grade is presented in Table 1. The participants range from K7 (first graders of junior high school) to K12 (third graders of senior high school). The native language of all the participants is Chinese, and they started learning English as a foreign language from fourth grade in primary schools at the age of 9 or 10. Their involvement in this research is indirect in the sense that only their compositions written in English are collected and analyzed.

**Table 1:** Participants’ Basic Information.

Grade	No	Age
K7 (first grade of junior high school)	75	12–13
K8 (second grade of junior high school)	61	13–14
K9 (second grade of junior high school)	69	14–15
K10 (first grade of senior high school)	78	15–16
K11 (second grade of senior high school)	74	16–17
K12 (third grade of senior high school)	79	17–18

### Materials

Our self-built corpus contains 436 English compositions written by the above-mentioned participants within the prescribed time limit in the class, with a total of 55,500 words. The compositions collected are narrative. The topics of the compositions are basically about their own experiences, such as my weekend, an embarrassing experience, an unforgettable experience, an annoying experience, etc. We controlled the genre and the subject matter by assigning compositions of similar topics to make a better longitudinal comparison. The contrastive corpus of native English was extracted from FLOB, a British written English corpus, containing articles from fifteen fields, which can be the representative of articles of written English. We selected the category of general fiction in FLOB as our contrastive corpus, because this category is most similar to the narratives we collected. The contrastive corpus has 56,600 words in total, which is very close to our learner corpus.

Linguistic Inquiry and Word Count, or LIWC, is a text analysis application, originally developed to provide an efficient and effective method for studying the various emotional, cognitive, and structural components present in individuals’ verbal and written speech samples [26]. LIWC has been increasingly used to analyze language use from a psychological perspective. People’ thinking patterns can be reflected in their writing, thus, LIWC is also appropriate for analyzing Chinese students’ EAT demonstrated by their writings in English.

### Procedure

Thinking can vary in depth and complexity, which is reflected in the words people use to connect thoughts [27]. Pennebaker [28] found out three factors that pointed to very different writing styles—formal, analytic, and narrative—by categorizing various classes of function words and using a statistical method called factor analysis. More specifically, the analytic thinking factor, reflecting cognitive complexity, identifies people who make efforts in parsing their world. People with high analytic thinking read more and have more complex views of themselves than those with low analytic thinking. Words that embody analytic thinking include exclusives, negations, causal words, insight words, quantifiers, tentative words and certainty words. People of high analytic thinking apply high frequency of the above seven dimensions of words in their writing.

Exclusive words include “but”, “without”, “exclude”, etc. Exclusive words are used when people are attempting to make a distinction between what is in a category and what is not [27]. As for negations (e.g. no, not, never), they are also necessary for making distinctions. Thus, people who are analytic or categorical thinkers tend to use negations when describing an event [28]. Causal words (e.g. because, effect, hence) and insight words (e.g. think, know, consider) are two dimensions of cognitive mechanisms that are indicative of a more complex language. Besides, quantifiers (e.g. few, many, much) also belong to the words signifying greater cognitive complexity and detailed information. Tentative words (e.g. maybe, perhaps, guess) are used when people are uncertain or insecure about what they say, so higher use of tentative words might suggest that a participant has not yet processed an event and formed it into a story. On the contrary, the use of certainty words (e.g. absolutely, always, never) indicates that the participants recounting the story feel certain about what they are writing. According to their functions, the seven dimensions are divided into three aspects: the ability of making distinctions (exclusive words and negations), the degree of cognitive complexity (causal words, insight words and quantifiers) and the degree of thinking activeness (tentative words and certainty words).

The learner corpus we created is analyzed by LIWC2007 in order to find out whether English language learning can foster Chinese teenagers’ EAT. Since the corpus covers 6 grades, we will also be able to observe EAT’s developmental path of each grade through different LIWC dimensions. This will help answer the first research question.

To answer the second research question—to what extent can their EAT be developed, the contrastive corpus of FLOB is used. By comparing the variables in the learner corpus and the contrastive corpus, we will be able to tell how well the EAT of Chinese teenagers fare, and where the room for improvement lies in.

Finally, the data are analyzed as described in **Data analysis**.

### Data analysis

To investigate Chinese students’ developmental path of EAT during across six grades, LIWC2007 was used to calculate seven dimensions of each student’s English composition. LIWC has two main components: programs and dictionaries. As the core, dictionaries define the categories and the word lists. The programs compare words in the inputted texts with those in dictionaries and then output word frequencies of different dimensions, presented in percentages [29]. Then the means of seven dimensions of six grades were calculated using SPSS 20.0, which enabled us to see the overall development of students’ EAT. To examine whether the variations of seven dimensions with grades were significant, ANOVA was conducted. Then, the seven dimensions were divided into three aspects according to their function, namely, the ability of making distinctions, the degree of cognitive complexity and the degree of thinking activeness. In the same vein, ANOVA was conducted to investigate whether the variations of the three aspects were significant. All this helped to answer Question 1.

To compare K12 students’ EAT with English native speakers’ analytic thinking, independent T-test was conducted to see whether there exist significant differences in the two groups’ use of seven dimensions. To investigate why Chinese K12 students’ used more causal words and insight words than native speakers (other dimensions being fewer), we searched all the words belonging to these two dimensions listed in LIWC2007 word category file and compared their frequencies and uses in the self-built corpus of narrative writing of K12 students with the contrastive corpus (the sub-corpus of FLOB) through AntConc 3.4.3. Finally, Chi-square test was conducted to investigate whether the frequencies of certain words, such as “because”, are significantly different between K12 students and native speakers. As one would expect, this process would answer the Question 2.

## Results and Discussions

To answer the question that whether learning English can foster Chinese learners’ EAT, we will closely examine the data reflecting the development of EAT of high school students across six consecutive grades. Related results and discussions of the developmental path of EAT are presented in the following sections. Their EAT is discussed from the following three aspects: the ability of making distinctions, the degree of cognitive complexity and the degree of thinking activeness. In order to find out to what extent their EAT has developed, we will compare the data we extracted from K12 students with those from contrastive corpus, focusing on causal words and insight words, whose uses are different from the other five dimensions.

### The developmental path of EAT demonstrated by Chinese students’ writings in English

If the EAT of Chinese EFL learners improves along with the improvement of English language ability, then the percentages of seven dimensions reflecting the EAT will show increasing tendencies along with the increases of grades. It has been found that Chinese senior high school students’ lexical competence in English writing improves significantly with the increase of their grades [30], so it is reasonable to hypothesize that Chinese EFL learners’ overall language ability also improves by grades. Table 2 shows the average percentages of seven LIWC2007 dimensions of the 6 grades.

**Table 2:** The Means of the Seven LIWC Dimensions.

		Grades					
Variables of Analytic Thinking	Examples	K7	K8	K9	K10	K11	K12
1. Insight	Think, know, consider	0.84	0.91	2.05	2.95	2.99	2.8
2. Causation	Because, effect, hence	0.49	0.69	0.83	1.41	1.54	1.52
3. Tentative	Maybe, perhaps, guess	0.75	0.78	1.61	1.96	1.82	2.28
4. Certainty	Always, never	0.54	0.29	0.66	1.06	1.21	0.99
5. Exclusive	But, without, exclude	0.91	1.2	1.65	2.27	2.08	2.31
6. Negations	No, not, never	0.62	0.86	0.93	2.04	1.83	2.11
7. Quantifiers	Few, many, much	0.9	1.36	2.14	1.91	1.91	1.99

As can be seen, the tendencies of increase by grades were observed in all dimensions, which indicates the improvement of students’ EAT demonstrated by their English writings. The results of one-way ANOVA analysis show that the percentages of seven dimensions of different grades are all significantly different (Insight: F(5, 430) = 35.530, *p*<0.01; Causation: F(5, 430) = 15.235, *p*<0.01; Tentative: F(5, 430) = 17.140, *p*<0.01; Certainty: F(5, 430) = 10.351, *p*<0.01; Exclusive: F(5, 430) = 14.812, *p*<0.01; Negations: F(5, 430) = 25.650, *p*<0.01; Quantifiers: F(5, 430) = 8.314, *p*<0.01). This is consistent with our prediction that the EAT of Chinese EFL learners improves with the improvement of their English ability. To save time and space, the seven dimensions are divided into three aspects and discussed aspect by aspect, instead of dimension by dimension.

#### The ability of making distinctions

**Fig 1:**
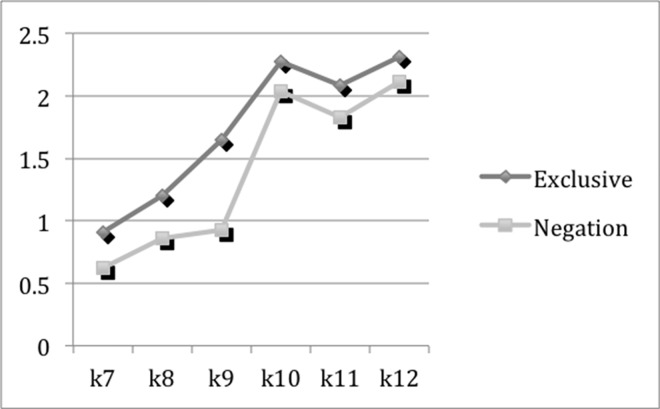
The Means of Exclusive Words and Negations.

Exclusive words and negations are two dimensions that demonstrate the ability of making distinctions. Their means of the 6 grades are presented in Fig 1, which indicates that exclusive words and negations have consistently high variation tendencies. Their changes by grades present a mild but significant growth (*p*<0.01) though experiencing a decrease from K11 to K12. As mentioned above, both exclusive words and negations are used when people are making distinctions. Moreover, as Newman, Pennebaker, Berry and Richards [31] pointed out, exclusive words are used at a higher frequency among people processing cognitively complicated tasks and adding information about the stories. So it can be seen that Chinese students’ ability of making distinctions between what is in a category and what is not has improved significantly in their English writing during high school period—which spans six years. The ability of making distinctions is essential in analytic thinking.

#### The degree of cognitive complexity

**Fig 2:**
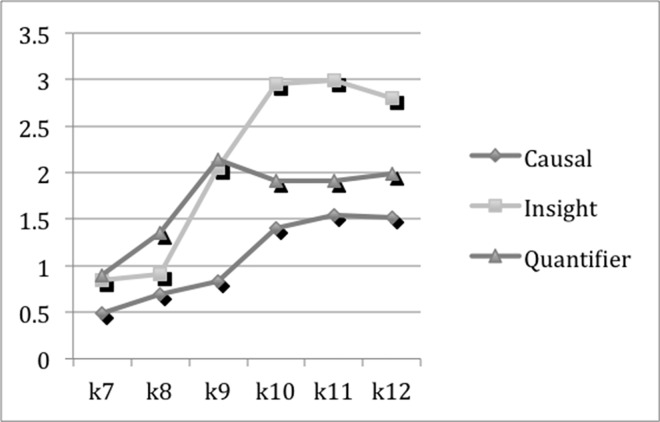
The Means of Causal, Insight Words and Quantifiers.

Causal and insight words are intended to capture the degree to which participants are thinking actively in their writing. The causal words are used when people are attempting to put together causes and reasons for the events and emotions they are describing. The insight words reflect the degree to which individuals are referring specifically to cognitive processes associated with thinking [32]. Causal words, insight words, as two subcategories of cognitive mechanism [33], and quantifiers [28], are indicatives of words signifying greater cognitive complexity. Fig 2 presents the means of causal, insight words and quantifiers. As shown in Fig 2, the peak of insight words and casual words both appear at K11, but the mean of quantifiers reaches its highest at K9, and then experiences curvilinear changes. Although the peaks of these three dimensions do not lie at K12, there are significant (*p*<0.01) progresses by grades.

#### The degree of thinking activeness

**Fig 3:**
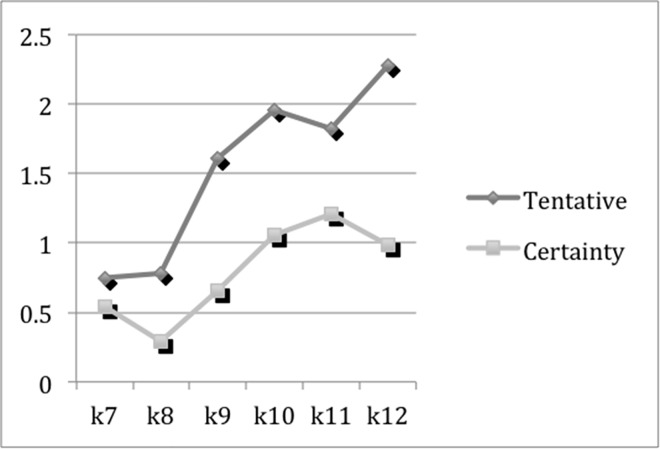
The Means of Tentative and Certainty Words.

Tentative words are used when people are uncertain of what they are writing and certainty words indicate that the participants feel certain about what they are writing. Previous study [34] shows that undisclosed events are more tentative than disclosed event, but not less certain. When people are recounting an undisclosed event, more active thinking and cognitive process are needed. Fig 3 shows the changes of tentative and certainty words by grades. Though tentative words witness a slight decrease from K10 to K11 and certainty words decrease from K7 to K8 and from K11 to K12, their increases by grades are significant (*p*<0.01). The increases of both tentative words and certainty shown in Fig 3 indicate that students are thinking more and more actively when they are recounting an event in narrative thinking.

**Fig 4:**
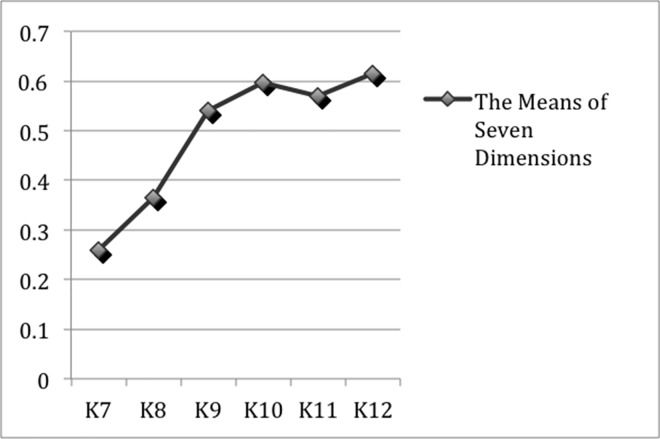
The Variation Tendency of Seven Dimensions.

To better see the developmental path of Chinese students’ EAT, we calculated the means of seven dimensions altogether for every grade and obtained the variation tendency of Chinese students’ EAT in Fig 4. As can be seen from the figure, the tendency of increase by grades is obvious in the seven dimensions, which enables us to see the dynamic improvements of students’ EAT across six different grades.

The quantitative analysis of the self-built corpus indicates that the figures of seven dimensions reflecting EAT have all increased, which answers Question 1, and proves that learning English as a foreign language does foster Chinese learners’ EAT.

#### The factors contributing to the improvement of Chinese students’ EAT and GAT

From the results of data analysis and discussions above, it can be concluded that learning English as a foreign language does foster Chinese learners’ EAT, and their EAT develops with the increase of their language proficiency. Admittedly, it is not only the improvement of their English language proficiency but also the increase of their age that contributes to the improvement of Chinese students’ EAT.

First, Chinese learners’ improvement of English language ability can contribute to their improvement of EAT. According to the principle of linguistic relativity, the linguistic categories and usage affect the ways in which its respective speakers conceptualize their worldviews, or otherwise influence their cognitive processes [4]. English is a language that is very different from Chinese in sentence structures, which can be perceived as different representations of analytic thinking and holistic thinking respectively. From the perspective of cultural psychology, in terms of thinking styles, it is widely accepted that East Asians hold a holistic assumption that every element in the world is somehow interconnected, whereas Westerners tend to view the universe as composed of independent objects [35, 36]. The advantages of learning a foreign language go beyond the acquisition of language itself, they can involve the shaping of thinking styles. The switching hypothesis proposes that bilingual children develop a more flexible learning set as a result of switching languages and making use of two different perspectives [37]. Ramirez and Price-Williams [38] suggested that speaking two languages and belonging to two cultures foster some kind of “bicognitivity”; that is, “in the same way that the bilingual child switches language codes in response to the demand characteristic of the socio-linguistic situation, so the bicognitive child switches cognitive styles as demanded” [39]. The different activation patterns in cognitive control mechanism have been observed for language switching against different baseline conditions in an fMRI study of English-Chinese late bilinguals [40], which shows that bilinguals adopt different cognitive styles when switching languages. When Chinese EFL learners are writing English compositions, they switch their cognitive style from holistic thinking to analytic thinking based on the language most active in their minds when they switch from Chinese to English. In this sense, our research has extended Duncan and DeAvail’s finding by showing that not only bilingual children, but also teenage EFL learners, who learn English in the Chinese environment, are also capable of switching their cognitive styles as demanded.

Besides the improvement of English language ability, Chinese students’ EAT is also affected by the increase of their age. Early in Pennebaker and Stone’s [41] research, it is shown that individuals demonstrate a general pattern of increasing cognitive complexity at teenager stage. The researchers collected text samples from over 3000 research participants ranging in age from 8 to 70+ and analyzed how age affects individuals’ emotional experience and expression, identity and social relationship, time orientation and cognitive abilities. Their results show that causal words (0.84–1.10), insight words (1.40–2.28) and exclusive words (3.35–3.98) all increased from the age brackets of 8–14 and 15–24, which is consistent with the variation tendencies of causal words (0.49–1.52), insight words (0.84–2.80) and exclusive words (0.91–2.31) in our current Chinese students’ English writings. From K7 to K12, there is an age difference of 6 in our subjects, and these six adolescent years is critical for fostering cognitive abilities. It is evident that the six years have also helped our subjects deepen their cognitive complexity. As they grow older, their thinking ability becomes mature gradually and their cognitive abilities in English improve, which is reflected in the seven dimensions of cognitive category in their writings.

As bilinguals mastering Chinese and English, will Chinese students’ EAT influence their GAT? It is reasonable to assume that there exists “Thinking Ability Transfer” during the acquisition of a foreign language. Since it is the same person (brain) that picks up two languages simultaneously, the ability of EAT will automatically and unconsciously impact Chinese students’ cognitive processes and help improve their GAT. Likewise, the increase of age has a positive effect on GAT, because as students grow older, their thinking ability becomes mature, which we have already discussed above.

In the above section, we explored the developmental process of the EAT of Chinese junior and senior high school students, whose language competence and intelligence are at the critical developmental stage. It is proved that Chinese teenagers’ EAT can be fostered through English learning, and it develops with the increase of English proficiency and age. This is a cross-sectional comparison, and the subjects are homogenous. It would be interesting to see to what extent their EAT has developed, or how much room they have for improvement in terms of EAT. These questions can only be answered through a comparison between our learner corpus and native speakers’ corpus.

### The comparison of Chinese EFL learners’ EAT and that of native English speakers

The following section will address Question 2: To what extent has Chinese EFL learners’ EAT developed? Will their analytic thinking reach the level of English native speakers? If the answer is negative, what are the main problems and how much room for improvement do Chinese EFL learners have?

#### The overall description of the analytic thinking of Chinese K12 students and native speakers

**Fig 5:**
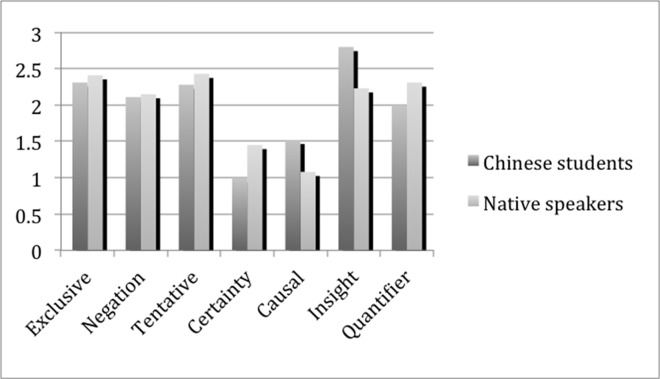
Comparison Between Chinese K12 Students and Native Speakers.

K12 is the last year of high school education in China, and the students are normally 17–19 years old. After six years’ English learning, their English language ability reaches the peak during high school period. After entering colleges, they usually spend less time and efforts in English learning, and more in the specialized courses. According to Pennebaker’s [40] research results, the means of causal words (1.10), insight words (2.28) and exclusive words (3.9) reach the highest at the age bracket of 15–24, which implies that the peak of cognitive complexity is at the age bracket of 15–24. All indexes of cognitive markers (causal words, insight words and exclusive words) increase appreciably from childhood to early adolescence, and then remain relatively stable between age 15 and 69. Hence, it is viable to compare the EAT of K12 Chinese students with that of English native speakers. Fig 5 shows the average percentages of seven dimensions of K12 Chinese students and English native speakers.

As shown in Fig 5, it is obvious that compared with English native speakers, Chinese students of K12 used fewer exclusive words, negations, tentative words, certainty words and quantifiers, while more causal words and insight words in narrative writing. The results of independent T-test in Table 3 show significant differences in Chinese students’ and English native speakers’ use of these dimensions, except negations. Compared with native speakers, Chinese K12 students used significantly fewer exclusive words, tentative words, certainty words and quantifiers, but significantly more causal words and insight words. There was no difference in the use of negations. These results might suggest that though Chinese K12 students’ EAT has developed significantly, it has not reached the level of native speakers. Among the seven dimensions, only two dimensions—causal words and insight words—presented the opposite statistical analysis results, that is, Chinese K12 used more causal and insight words. Why is so? Further statistical analysis has provided some possible explanations, which will be discussed in the following section

**Table 3:** The Results of Independent T-test between Chinese K12 Students’ Uses of Seven Dimensions and Those of Native Speakers.

	Exclusive		Negation		Tentative		Certainty		Causal		Insight		Quantifier	
	F	*p*	F	*p*	F	*p*	F	*p*	F	*p*	F	*p*	F	*p*
**Chinese K12 & Native speakers**	5.05	0.027	3.34	0.07	9.40	0.003	9.44	0.003	19.36	0.000	9.88	0.002	8.92	0.004

#### The comparison of Chinese K12 students and native speakers’ uses of causal words and insight words

We searched all the words belonging to the dimensions of causal words and insight words listed in LIWC2007 word category file [42] and compared their frequencies and uses in the self-built corpus and the contrastive corpus. Through AntConc 3.4.3, it is found that there were 660 frequencies of 32 different causal words in the learner corpus and 552 frequencies of 56 different causal words in the contrastive corpus. It can be seen that the causal words in the contrastive corpus are much more diversified than those in the corpus of students’ writings. Table 4 shows the ten most frequent causal words in the two corpora.

**Table 4:** Ten Most Frequent Causal Words.

	Narrative Compositions (Learner Corpus)			FLOB General fiction (Contrastive Corpus)	
Causal Words	Frequencies	Percentages	Causal Words	Frequencies	Percentages
make	195	29.5%	make	139	21.1%
because	171	25.9%	how	71	10.8%
how	104	15.8%	because	59	8.9%
why	48	7.3%	why	57	8.6%
change	19	2.9%	use	37	5.6%
use	19	2.9%	since	30	4.5%
since	14	2.1%	change	16	2.4%
reason	11	1.7%	cause	13	2.0%
therefore	10	1.5%	lead	11	1.7%
result	6	0.9%	reason	10	1.5%
**Total**	597	91.4%	**Total**	443	80.3%

As shown in Table 4, the ten most frequent casual words in the two corpora are almost the same, but the percentages show striking differences. In the learner corpus, the ten most frequent causal words make up 91.4% and in the contrastive corpus, the percentage is 80.3%, which indicates that causal words used by native speakers are richer than Chinese students. As for the three most frequent causal words, the difference is even greater. In the learner corpus, the three most frequent words make up 71.2% and in the contrastive corpus, the percentage is 48.7%. So, from the frequencies of causal words in the two corpora, it can be seen that compared with English native speakers, Chinese students used fewer different and less diversified causal words.

In the ten most frequent words, seven words are the same in the two corpora: make (made, makes, making), because, how, why, change (changed, changes, changing), use (uses, used, using) and reason (reasons). The results of Chi-square test analysis show that only the frequencies of “because” (χ^2^ = 45.291, *p*<0.01) and “use” (χ^2^ = 9.975, *p* = .002) are significantly different between Chinese students and native speakers. We searched all “because” in the learner corpus and examined whether they were properly used. The in-depth analysis shows that “because” were overused/misused in students’ writings. Among 171 “because”, 14 “because” were not used properly by Chinese students. For example, in text 0203:

*“In the last weekend, I sleep last, because I didn’t saw my mother.”

In text 0474:

*“I can't do well in my study because I have so many hobbies.”

There are no obvious causal relationships between the subordinate clauses and the main clauses.

Compared with the specific use of the words belonging to causal words, the words in insight words category are more complicated. Through corpus software, it was found that there are 1307 frequencies of 62 different insight words in the learner corpus and 1176 frequencies of 91 different insight words in the contrastive corpus. It can be seen that the insight words in the contrastive corpus are more diversified than those in the learner corpus. As shown in Table 5, the ten most frequent insight words in the two corpora are very different. In the learner corpus, the ten most frequent words make up 74.8% and in the contrastive corpus, the percentage is 66.9. In the learner corpus, the three most frequent words reach 45.9%, but the percentage is 39.1 in the contrastive corpus.

**Table 5:** Ten Most Frequent Insight Words.

	Narrative Compositions (Learner Corpus)			FLOB General fiction (Contrastive Corpus)	
Insight Words	Frequencies	Percentages	Insight Words	Frequencies	Percentages
feel	244	18.7%	know	209	17.8%
think	218	16.7%	think	176	15.0%
know	138	10.6%	seem	75	6.4%
find	124	9.5%	feel	74	6.3%
realize	54	4.1%	find	74	6.3%
remember	50	3.8%	remember	54	4.6%
learn	46	3.5%	mean	38	3.2%
lesson	39	3.0%	believe	38	3.2%
seem	35	2.7%	become	27	2.3%
become	30	2.3%	sense	22	1.9%
**Total**	978	74.8%	**Total**	787	66.9%

We searched the collocations involving “think (thinks, thought, thinking)” in two corpora because previous study shows that EFL learners overuse “think” because they use too many “I think” in compositions [43]. In our study, among 218 “think (thinks, thought, thinking)” used by Chinese students, 97 are stance markers “I think”, while native speakers only used 24 “I think” as stance markers. The data analysis shows that Chinese students (44.5%) use “I think” much more frequently than native speakers (13.6%). This result confirms the conclusion in previous studies that EFL learners, including Chinese learners, overuse “I think” as stance markers in their compositions [43, 44].

#### The factors contributing to Chinese students’ and native speakers’ differences of analytic thinking

Though Chinese students’ EAT has increased with the improvement of their English language competence and age, it has not reached the level of native speakers. Compared with English native speakers, Chinese students of K12 used significantly fewer exclusive words, tentative words, certainty words and quantifiers, not only in total number but also in diversity, while significantly more causal words and insight words in their narrative writings, in terms of absolute number but not in diversity. Further data analysis shows that Chinese students used more causal words and insight words than native speakers because they overused/misused some common words and collocations, such as “because” and “I think”.

On the one hand, the disparity of EAT in English between Chinese students of K12 and English native speakers is attributed to the difference of English proficiency, and on the other hand, the disparity is related to the age gap of Chinese students and the writers of general fictions. The Chinese students who wrote the compositions are only 17–19 years old, while the writers whose novels were selected and included in the general fiction in FLOB are well-known novelists who are much older than the Chinese students. Compared with younger people, the elder people are better able to describe their own experiences and their world, due to the increase of cognitive complexity and the gaining of wisdom [41].

There exist differences in Chinese learners’ EAT compared with that of native speakers, i. e. English native speakers are significantly better in the ability of making distinctions and the degree of thinking activeness. Although the results of data analysis show that Chinese K12 school students’ EAT has not yet reached the level of native speakers, Chinese students’ EAT has developed to a certain high level. First, on the uses of negations, casual words and insight words, Chinese K12 students are no worse than native speakers. Besides, Chinese students are at a disadvantage in terms of both English proficiency and age, which naturally resulted in the fact that native speakers have automatic advantages over Chinese students in cognitive complexity and wisdom. Moreover, Chinese students used foreign language to write, while native speakers are professional novelists who are proficient in their native language, English. Taking all the above facts into consideration, though Chinese K12 students’ EAT has not reached the level of native speakers, their EAT has improved greatly and to a satisfactorily high level.

Our study confirmed the positive influences of bilingualism on problem solving by discovering the bilingual advantages on the development of analytic thinking. Specifically, analytic thinking involves discrimination, in other words, the process of making distinctions [45]. The increases of exclusive words and negations with grades suggest that Chinese students’ ability of making a distinction improves along with their English language ability. This indicates that foreign language learning has positive effects on Chinese students’ ability of making distinctions in analytic thinking, but their abilities have not reached the level of native speakers manifested by the comparison between Chinese students’ and native speakers’ uses of exclusive words and negations. The complexity of language is, to a certain degree, the representation of the cognitive complexity, other factors being equal. Chinese students’ uses of causal words, insight words and quantifiers all experienced dramatic increases during the junior high school period, but remained in quite steady states during the senior high school period. Causality not only accounts for the use of causal relations and their linguistic expressions, but also for cognitive complexity of discourse connections in language acquisition and discourse processing [46]. Causal words, together with insight words [27, 32, 33, 41], and quantifiers [28], are indicatives of words signifying greater cognitive complexity. Cognitive complexity is a psychological variable that indicates how complex or simple is the perceptual skill of a person. A person who is measured high on cognitive complexity is more apt to perceive subtle differences. This indicates that Chinese students’ cognitive complexity improves significantly during the junior high school period, and comes to the plateau in the senior high school period. Our study also shows that Chinese students’ cognitive complexity is not lower than native speakers’ because Chinese K12 students used causal words and insight words more frequently than native speakers, though they overused some of the causal words and insight words.

## Conclusions and Implications

Our data and results suggest that learning English does foster Chinese learners’ EAT and this analytic proficiency improves along with the improvement of their English language proficiency and age, but has not reached the level of native speakers. It is postulated that this EAT might help improve learners’ GAT, though further research is needed.

The improvement of Chinese students’ EAT is reflected in three aspects: the improvements of the ability of making distinctions, the degree of cognitive complexity, and the degree of thinking activeness. Chinese students, who are characterized by holistic thinking, improve their EAT through learning English whose speakers’ are characterized by analytic thinking. The learning of English and the increase of age contribute to the improvement of their EAT. Moreover, we believe that due to the effect of Thinking Ability Transfer, the EAT that students have gained from learning English will unconsciously and subtly integrate with their GAT.

The comparison between Chinese learners’ EAT and native speakers’ shows that though Chinese students’ EAT has increased with the improvement of their English language proficiency and age, it has not reached the level of native speakers. The disparity of EAT between Chinese students of K12 and English native speakers is attributed to the differences in thinking styles caused by different native languages, the differences of English proficiency and the age gap between the Chinese students and the writers of general fictions. Taking the three reasons into consideration, though Chinese K12 students’ EAT has not reached the level of native speakers, their analytic thinking has improved greatly and to a satisfactorily high level.

The present study demonstrated the possibility of analyzing the writings by Chinese EFL learners to ascertain the positive influences of learning English on fostering Chinese high school students’ EAT. The improvement of Chinese learners’ EAT is believed to act on their general analytic thinking and thus have a positive impact on their analytic thinking unconsciously. In other words, EAT might have a positive transfer to Chinese students’ analytic thinking, and the EAT may be captured from their writings in Chinese. But to what extent the improvement of students’ EAT and the increase of their age positively influence their general analytic thinking proficiency and their analytic thinking of Chinese have not been touched upon in the present research. This can be our future research objective, which can be done by comparing Chinese who learn English with those who don’t. Another limitation is that our research is a cross-sectional study, and it will be more ideal if we could follow up the same group of Chinese EFL learners in our future research.

Moreover, our research has not only confirmed switching hypothesis but also extended Duncan and DeAvail’s finding by showing that not only bilingual children, but also adolescent EFL learners are able to switch their cognitive styles as demanded, because their English writings provide ample instances of analytic thinking instead of holistic thinking.

Finally, the present study has confirmed that bilingualism has cognitive benefits on the bilinguals by means of the acquisition of a foreign language and has provided evidence for Sapir-Whorf hypothesis that learning a foreign language can also have effects on shaping bilinguals’ thinking style, which further helps us understand the interrelation between language and human mind.
